# Epidemiological and genomic analysis of dengue cases in Guangzhou, China, from 2010 to 2019

**DOI:** 10.1038/s41598-023-28453-y

**Published:** 2023-02-07

**Authors:** Liyun Jiang, Yuan Liu, Wenzhe Su, Wenhui Liu, Zhiqiang Dong, Yuxiang Long, Lei Luo, Qinlong Jing, Yimin Cao, Xinwei Wu, Biao Di

**Affiliations:** 1 AIDS Control and Prevention Department, Guangzhou Centre for Disease Control and Prevention, Baiyunqu Qidelu 1, Guangdong, China; 2 Centre for Disease Control and Prevention of Liwan District of Guangzhou, Liwan Zhoumenxijie 32, Guangdong, China; 3 Virology Department, Guangzhou Centre for Disease Control and Prevention, Baiyunqu Qidelu 1, Guangdong, China; 4 Epidemiology Department, Guangzhou Centre for Disease Control and Prevention, Baiyunqu Qidelu 1, Guangdong, China; 5 State Key Laboratory of Organ Failure Research, Department of Biostatistics, Guangdong Provincial Key Laboratory of Tropical Disease Research, School of Public Health, Southern Medical University, Guangzhou, China; 6 Microbiology Department, Guangzhou Centre for Disease Control and Prevention, Baiyunqu Qidelu 1, Guangdong, China

**Keywords:** Molecular biology, Diseases

## Abstract

With a long epidemic history and a large number of dengue cases, Guangzhou is a key city for controlling dengue in China. The demographic information regarding dengue cases, and the genomic characteristics of the envelope gene of dengue viruses, as well as the associations between these factors were investigated from 2010 to 2019, to improve the understanding of the epidemiology of dengue in Guangzhou. Demographic data on 44,385 dengue cases reported to the Notifiable Infectious Disease Report System were analyzed using IBM SPSS Statistics v. 20. Dengue virus isolates from patient sera were sequenced, and phylogenetic trees were constructed using PhyML 3.1. There was no statistical difference in the risk of dengue infection between males and females. Unlike other areas in which dengue is endemic, the infection risk in Guangzhou increased with age. Surveillance identified four serotypes responsible for dengue infections in Guangzhou. Serotype 1 remained prevalent for most of the study period, whereas serotypes 3 and 4 were prevalent in 2012 and 2010, respectively. Different serotypes underwent genotype and sublineage shifts. The epidemiological characteristics and phylogeny of dengue in Guangzhou suggested that although it has circulated in Guangzhou for decades, it has not been endemic in Guangzhou. Meanwhile, shifts in genotypes, rather than in serotypes, might have caused dengue epidemics in Guangzhou.

## Introduction

Dengue fever is caused by the mosquito-borne dengue virus (DENV). It is endemic in subtropical and tropical regions worldwide. In 2019, the World Health Organization (WHO) listed dengue as one of 10 potentially threatening diseases. According to WHO data, the annual number of reported cases and the estimated annual incidence rate of dengue have remained above 200,000 and 10 per 100,000, respectively, in Southeast Asia, since 2001^1^. A total of 59,334 cases were reported in mainland China between 2005 and 2015. Not only has the incidence increased, but the geographical range has also expanded in recent years^2^.

DENV has circulated in Guangzhou since 1978^3^, and with the increasing threat of dengue, Guangzhou has become a key target city in the control of DENV in mainland China. In 2014, the number of dengue cases reported in Guangzhou constituted 80% of the national total^4,5^. From 2010 to 2019, Guangzhou experienced recurrent dengue epidemics, resulting in more than 40,000 reported cases. Dengue has posed a heavy burden on Guangzhou. Its humid subtropical climate, with a mean annual temperature, an annual precipitation, and a mean relative humidity of 24 °C, 1435.5 mm, and 84.2%, respectively, as well as the effects of urbanization, provide an appropriate environment for the DENV vector, *Aedes albopictus. Aedes aegypti* has not been identified in the area.

There are four serotypes of DENV, grouped by the antigenicity of the envelope protein, which plays a significant role in infection and epidemic dynamics. During infection, the envelope protein of DENV binds to host cell receptors, which then fuses with and penetrates host cells. It stimulates a host immune response by inducing production of specific protective and neutralizing antibodies. Antibody binding to the envelope protein inhibits cell attachment and membrane fusion, leading to virus neutralization^6,7^. The envelope gene, which harbors most molecular markers for pathogenicity, exhibits “hot spots” of higher mutation rates during epidemics. As the virulence and transmissibility differ between strains, the envelope gene is widely used to classify viruses into genetic groups (genotypes) within serotypes^8^.

We investigated the circulation dynamics of DENV over a 10-year period, complemented by an analysis of epidemiological data. We aimed to determine the impact of the four DENV serotypes on local epidemics, and to describe the spread of dengue in Guangzhou. This study provides insights into shifts in DENV genotypes, improves our understanding of the epidemiology of dengue between 2010 and 2019, and can help in designing policy and allocating prevention resources.

## Materials and methods

### Data collection

Dengue has been a legally notifiable disease in China since 1989, with mandatory timely reporting to the Notifiable Infectious Disease Report System (NIDRS). The present study analyzed cases reported to the NIDRS between 2010 and 2019. All cases analyzed here were diagnosed according to the Diagnostic Criteria for Dengue Fever (WS216-2008) issued by the Chinese Ministry of Health, which was revised to WS216-2018 in 2018. Dengue cases were defined as clinical manifestations in individuals with a history of travel to an epidemic area within the 14 days preceding the onset, or clinical manifestations with laboratory confirmation. Dengue cases with severe plasma leakage, bleeding, organ involvement, or shock were classified as severe dengue.

### Epidemiological and statistical analysis

Demographic data were obtained from the NIDRS. The annual incidence was calculated based on the number of cases and corresponding population size. Dengue cases were grouped by sex and age. Annual incidence rates were calculated for all specific age groups and specific sexes, using the corresponding annual number of residents as the denominator. The average annual rate of dengue-associated morbidity was estimated for each age group and for males and females using Negative Binomial regression analysis as follows:

$$Log(Case)=Offset(Log(Pop))+{\beta }_{0}+{\beta }_{1}*Year+{\beta }_{2}*Age+{\beta }_{3}*Sex$$

where $Case$ presents the annual incidence number of dengue fever for different combinations of age groups and sex groups in different years. $Pop$ refers to the annual population in the corresponding year. $Age$(i.e., 0–9, 10–19, 20–29, 30–39, 40–49, 50–59, 60–69 or > 69 years), $Sex$ (i.e., male or female) and $Year$ (i.e., from 2010 to 2019) were both defined as categorical variables.

The association at the multivariate level between the incidence rate of dengue and the independent variables were expressed as odds ratios (ORs) using a Negative Binomial regression model, where ORs > 1 indicated a higher risk compared to the reference group and ORs < 1 indicated a protective factor (lower risk) compared to the reference group. All statistical analyses were completed using R 4.0.5. Statistical significance was set at P < 0.05.

### Virus strains and sequences

From 2010 to 2018, patient sera were tested using real-time polymerase chain reaction (PCR). Dengue virus (DENV) strains were isolated from the positive samples. The *Aedes albopictus* clone C6*/*36 (ATCC^®^ CRL-1660™) cell line was purchased from the Cell Bank of the Chinese Academy of Sciences (Shanghai, China). C6/36 cell monolayers were inoculated with serum samples that had been diluted 50-fold with RPMI-1640 (Life Technologies Corporation, Grand Island, NY, USA) and incubated at 28 °C for 7 days. Cytopathic effects (CPE) were verified using indirect immunofluorescence (IF) tests. Cell cultures without CPE were inoculated into new C6/36 monolayers. Inoculated cells without CPE after three generations were considered negative. Supernatants of positive cultures were stored at − 80 °C.

During 2019, RNA extracted from serum samples was used to conduct RT-PCR and E gene sequencing directly, without virus isolation. Therefore, more sequences were obtained in 2019. This method is recommended for research because it provides better quality and replicability of the sequence results^9^.

We extracted RNA from cell culture supernatants or serum samples and conducted RT-PCR using the primers listed in Table S1. The products were used to sequence the E gene of DENV using Sanger sequencing. All the sequences were submitted to GenBank.

### Phylogenetic analysis

Sequences acquired in Guangzhou and reference sequences downloaded from GenBank were used to construct a maximum likelihood (ML) phylogenetic tree, using PhyML 3.1^10^. The model was selected by Smart Model Selection based on the Bayesian Information Criterion^11^. The fast likelihood-based method of eBayes was applied because of the large number of sequences. The genotypes of each serotype were grouped based on the criteria defined by Rico-Hesse^8^.

### Ethics approval and consent to participate

The study was conducted in accordance with the Declaration of Helsinki and approved by the Ethics Committee of the Guangzhou Centre for Disease Control and Prevention (protocol code GZCDC-ECHR-2021P0074). Written informed consent was obtained from all participants in the study, or from the parents or guardians of participants aged younger than 18 years.

## Results

### Descriptive analysis

**Figure 1:**
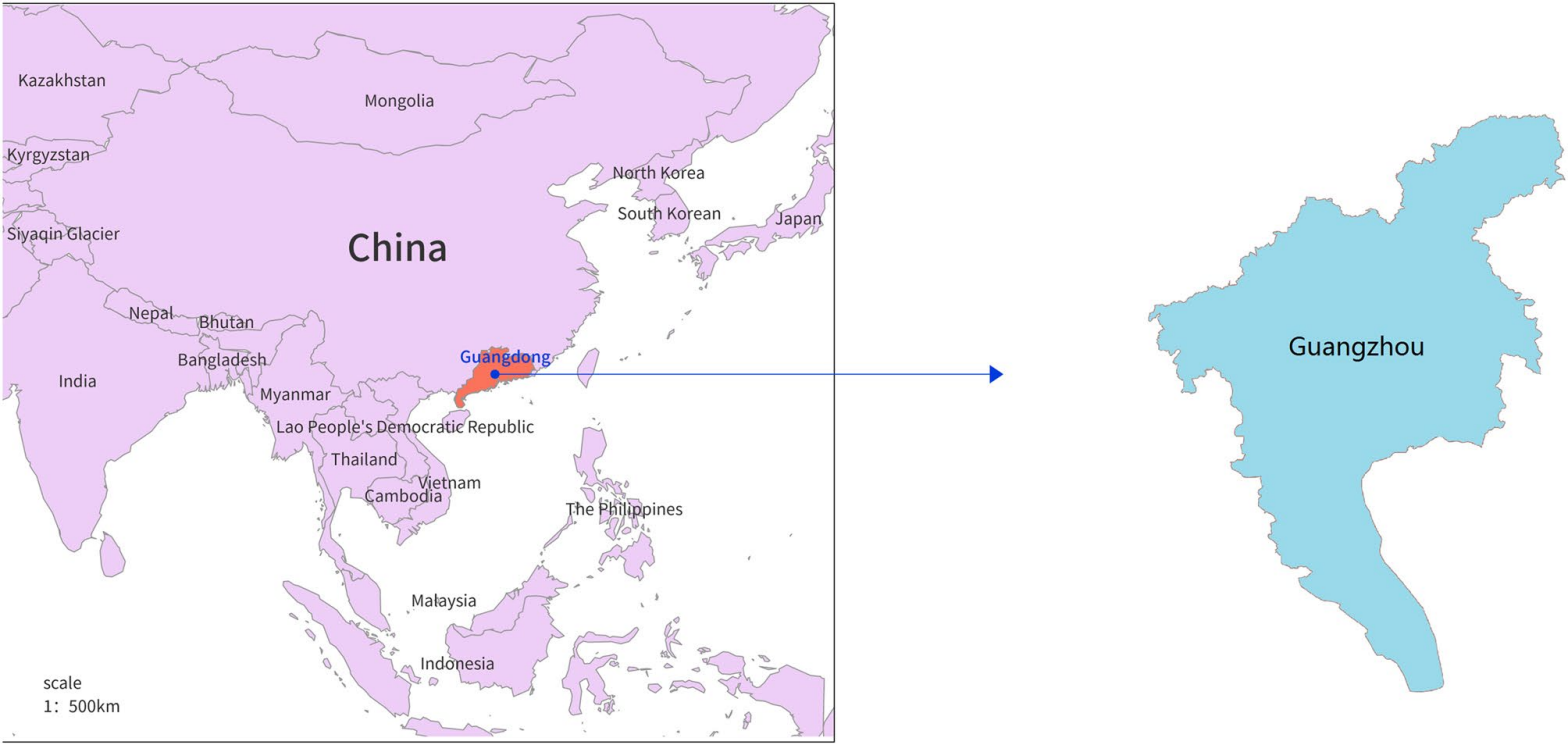
The location of Guangzhou. The blue dot indicates the location of Guangzhou in China. The map was created using ArcGIS9.3, URL: http://www.esrichina-bj.cn.

Guangzhou, with 15.30 million permanent residents in 2019, according to the Guangzhou Municipal Bureau of Statistics, is the capital city of Guangdong Province. It is located on the southeast coast of China, 112° 57–114° 3 E, and 22° 26–23° 56 N, covering an area of 7,434.40 km^2^. The location of Guangzhou is displayed in Fig. 1.

Between 2010 and 2019, there were 44,385 dengue cases in Guangzhou, of which 0.89% (397 cases) were classified as severe dengue and 0.02% (10 cases) resulted in death. Table 1 shows the annual incidence rates and incidence of dengue according to age and sex. The incidence rate gradually increased between 2010 and 2013, and peaked in 2014, with an incidence rate of 295.51/100,000 population. The incidence rate dipped in 2015 and then continued to rise.

**Table 1:** Incidence of dengue in Guangzhou between 2010 and 2019, according to age and sex.

Year	2010	2011	2012	2013	2014	2015	2016	2017	2018	2019	Total
Total cases	81	58	157	1270	38,036	137	283	1016	1422	1925	44,385
Dengue incidence rate*	0.73	0.46	1.23	9.89	295.51	1.06	2.1	7.23	9.81	12.92	–
Sex											
Male	44	26	72	606	18,743	77	144	530	802	1085	22,129
Female	37	32	85	664	19,293	60	139	486	620	840	22,256
Age (years)											
0–9	0	1	1	20	1750	1	6	33	29	58	1899
10–19	4	5	10	96	2771	6	14	77	75	112	3170
20–29	19	16	28	292	8135	35	67	232	344	440	9608
30–39	17	18	25	244	6984	40	55	206	315	435	8339
40–49	16	9	30	258	6586	17	54	165	251	359	7745
50–59	11	8	24	185	5117	18	37	136	177	261	5974
60–69	7	1	16	93	3575	13	28	85	142	149	4109
> 69	7	0	23	82	3118	7	22	82	89	111	3541

*Dengue incidence rate per 100,000 population.

The association at the multivariate level (Table 2) also indicates that the risk of dengue infection increased between 2010 and 2014, followed by a decrease in 2015 to similar levels as those seen in 2010, and then a steadily rising risk afterwards. The risk of infection was independent of gender. Although 57.88% (25,692/44,385) of the total cases occurred in the 20–49 years age group, after adjustment by gender and year, the infection risk showed an increasing trend with age. The annual incidence rates standardized by age and sex are shown in Table S2.

**Table 2:** Risk factors for dengue fever in Guangzhou from 2011–2019 at the multivariate level.

Factors	OR	95%CI	*P *value
Sex			
Female	**Ref**	**Ref**	**Ref**
Male	0.996	0.915–1.084	0.931
Age (years)			
0–9	**Ref**	**Ref**	**Ref**
10–19	1.628	1.339–1.978	< 0.001
20–29	2.601	2.162–3.131	< 0.001
30–39	3.089	2.565–3.721	< 0.001
40–49	3.083	2.561–3.714	< 0.001
50–59	3.477	2.885–4.191	< 0.001
60–69	3.934	3.245–4.770	< 0.001
> 69	3.726	3.066–4.530	< 0.001
Year			
2010	**Ref**	**Ref**	**Ref**
2011	0.589	0.406–0.849	0.005
2012	1.676	1.238–2.284	0.001
2013	12.985	10.004–17.035	< 0.001
2014	436.395	338.369–569.200	< 0.001
2015	1.364	1.001–1.869	0.051
2016	2.747	2.070–3.676	< 0.001
2017	9.680	7.445–12.718	< 0.001
2018	12.427	9.583–16.289	< 0.001
2019	16.378	12.647–21.442	< 0.001

*CI* confidence interval, *OR* odds ratio, *Ref*. reference group.

### Sequences and phylogenetic tree reconstruction

All the DENV sequences acquired in Guangzhou between 2010 and 2019 were deposited in the GenBank repository with accession numbers as shown in Table S3. Among these acquired sequences, 693, 142, 49, and 20 were of serotypes 1 (DENV1), 2 (DENV2), 3 (DENV3), and 4 (DENV4), respectively. All four serotypes were identified in Guangzhou over the study period, but co-circulation of all four serotypes was only documented in 2010, 2016, and 2018 (Table 3). DENV1 was the most frequently detected serotype throughout the decade, except in 2010 and 2012, when DENV4 and DENV3, respectively, were the most frequently detected serotypes. The sequences of DENV 1–4 detected in Guangzhou were distributed randomly among the branches of the phylogenetic trees. In a given year, individual sequences were shown to be derived from different genotypes or lineages; this suggests that these sequences may have had various origins. Meanwhile, over the 10-year period, no notable succession branch consisting of sequences from different years was seen in the phylogenetic trees.

**Table 3:** Annual numbers of sequences of each dengue virus serotype isolated in Guangzhou between 2010 and 2019.

Year	2010	2011	2012	2013	2014	2015	2016	2017	2018	2019	Total
DENV1	6	5	6	46	96	10	29	41	51	403	693
DENV2	2	0	2	2	10	9	24	16	39	38	142
DENV3	6	0	15	0	0	1	1	0	4	22	49
DENV4	14	0	0	0	0	0	1	0	5	0	20

**Figure 2:**
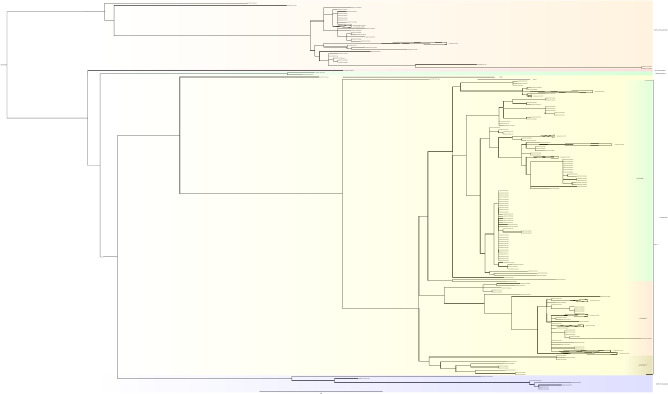
Phylogenetic trees of DENV1 E gene sequences. The reference sequences are indicated with black triangles for clarity, with the accession number, and year and country of isolation. Sequences detected in Guangzhou between 2010 and 2019 are indicated with the accession number, year of isolation, and the laboratory number. Some branches, including sequences from 1 year, are collapsed for clarity. A more detailed phylogenetic tree is shown in Figure S1.

A phylogenetic tree of DENV1 was constructed using 693 sequences from Guangzhou and 24 reference sequences from Genbank (Fig. 2). A more detailed phylogenetic tree is shown in Figure S1. The DENV1 sequences from Guangzhou were clustered into the Asia (n = 594), South Pacific (n = 4), and America/Africa (n = 95) genotypes. The Thailand and Malaysia genotypes were not identified during the study period. The Asia genotype was the most frequently detected genotype during all years, except 2014. The South Pacific genotype was only detected in 2010 (n = 2) and 2019 (n = 2). Sequences detected in 2014 and 2019 accounted for 68.42% (n = 65) and 20.00% (n = 19) of the America/Africa genotype, respectively. Of the 96 strains isolated in 2014, 65 (67.71%) were of the America/Africa genotype. The Asia genotype can be divided into three lineages: Asia-1, -2, and -3. Asia-3 contains all the Asian sequences detected in Guangzhou, which could be subdivided into sublineages I, II, and III.

**Figure 3:**
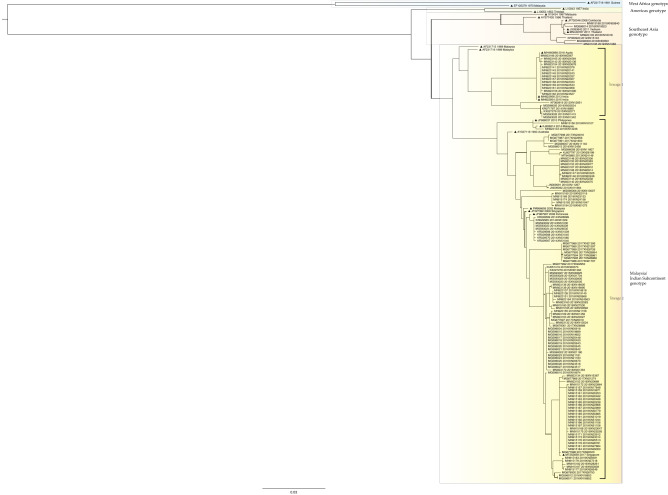
Phylogenetic tree of DENV2 E gene sequences. The reference sequences are indicated with black triangles for clarity, with the accession number, and year and country of isolation. Sequences detected in Guangzhou between 2010 and 2019 are indicated with the accession number, year of isolation, and the laboratory number.

A phylogenetic tree of DENV2 was constructed using 142 sequences from Guangzhou and 21 reference sequences from Genbank (Fig. 3). The DENV2 sequences from Guangzhou were clustered in the Malaysia/Indian subcontinent (n = 136) and Southeast Asia (n = 6) genotypes. The Malaysia/Indian subcontinent genotype was subdivided into lineages 1 and 2. No America or West Africa genotypes were identified.

**Figure 4:**
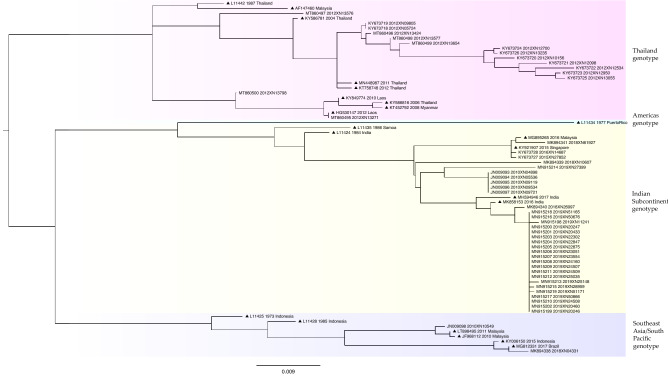
Phylogenetic tree of DENV3 E gene sequences. The reference sequences are indicated with black triangles, for clarity, with the accession number, and year and country of isolation. Sequences detected in Guangzhou between 2010 and 2019 are indicated with the accession number, year of isolation, and the laboratory number.

A phylogenetic tree of DENV3 was constructed using 49 sequences from Guangzhou and 22 reference sequences from Genbank (Fig. 4). Three genotypes of DENV3 were identified in Guangzhou: the Indian (n = 32), Thailand (n = 15), and Southeast Asia/South Pacific (n = 2) genotypes. All 15 sequences detected in 2012 were clustered in the Thailand genotype, which was not detected in the other years.

**Figure 5:**
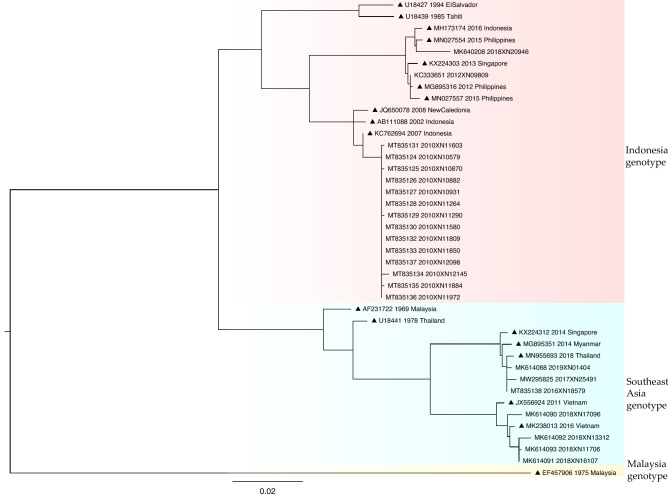
Phylogenetic tree of DENV4 E gene sequences. The reference sequences are indicated with black triangles for clarity, with the accession number, and year and country of isolation. Sequences detected in Guangzhou between 2010 and 2019 are indicated with the accession number, year of isolation, and the laboratory number.

A phylogenetic tree of DENV4 was constructed using 20 sequences from Guangzhou and 18 reference sequences (Fig. 5). Two genotypes of DENV4 were identified in Guangzhou: the Indonesia (n = 15) and Southeast Asia (n = 5) genotypes. The Malaysia genotype was not detected. The Indonesia genotype was detected in 2010 (14/15, 93%), with the exception of one sequence that was detected in 2018.

## Discussion

Dengue virus remained in circulation in Guangzhou between 2010 and 2019. As the incidence rate fluctuated, outbreaks occurred in a cyclical pattern approximately every 5 years, which is similar to the results of previous studies conducted in Guangzhou^12,13^ and other Chinese cities^14,15^. Although some researchers have reported the persistence of immunoglobulin G (IgG) for decades after dengue infection^16,17^, a serosurvey of antibodies against DENV after an epidemic in Nanning, China, revealed that IgG remained high for the following 3 years and decreased in the fourth year^18,19^, which may lead to a reduced level of protection. This may account for the cyclical pattern of outbreaks in Guangzhou every several years. Longitudinal studies of antibody levels among residents in Guangzhou must be conducted to determine whether the epidemics coincide with a decline in antibody levels.

In the present study, we performed statistical analyses of dengue virus incidence over a 10-year period, taking gender and the year of infection into consideration. No statistically significant difference in the risk of infection between males and females was observed at the multivariate level. This is not in line with previous studies, which have shown that rate of infection in males is significantly higher than that in female human hosts^12,20–22^. As shown in Table S2, the age-sex standard incidence rates varied over time in Guangzhou; the rates of male infections exceeded those of females in 2010, 2017, 2018 and 2019. However, the different research periods may have contributed to this difference. Meanwhile, studies have revealed that the majority of imported dengue cases in Guangzhou are in males^13,23^. But, with the surge in locally acquired cases, a high portion of infections were acquired at home or in public areas^4,24^, which may lead to the inconsistencies between studies.

Three age groups with ranges from 20 to 49 years contributed to more than half of dengue cases in Guangzhou, which is consistent with previous research^2,12,22^. However, the increased risk with age (Table 2) shown in the present study contrasted research findings from endemic countries^25–27^. This may be because infections in childhood and young adulthood lead to a high seroprevalence of dengue antibodies among older adults in endemic areas, which can protect them from reinfection and decrease the risk of infection in the older populations. These results indicate that the older age groups in Guangzhou were even more susceptible to DENV and provide evidence that dengue had not been endemic in Guangzhou. Meanwhile, DENV1-4 E gene sequences isolated in Guangzhou scattered in phylogenetic trees (Figs. 2, 3, 4, 5), displayed no sign of succession, and showed close relationships with Southeast Asian strains; this was in line with the results of complete genome research in Guangdong^5^, supporting the idea that dengue in Guangzhou is more likely caused by imported cases rather than being endemic.

The dominant serotype in Guangzhou in most years during the study period was DENV1, which has been the dominant serotype in Guangzhou since 2001^20,28^. However, DENV4 and DENV3 were the dominant serotypes in 2010 and 2012, respectively, and the incidence of infections with both serotypes was very low during subsequent years. DENV2 was detected every year except in 2011, but was never the predominant serotype during the study period. Antibodies against certain serotypes can protect against secondary infection from the same homotype. Therefore, the prevailing serotype cannot continue to circulate efficiently among populations. However, the large permanent and migrant populations of Guangzhou, which comprised 15.31 million and 9.67 million, respectively, at the end of 2019 according to data from the Guangzhou Municipal Civil Affairs Bureau, may account for the persistence of DENV1 as the dominant serotype during the study period. Further studies are needed to determine whether this can be explained by competition among serotypes, the sensitivity of local populations to different serotypes, or other factors.

As the prevailing serotype, DENV1 accounted for more than 76% of the identified sequences. The E gene phylogenetic tree of DENV1 showed that the Asia genotype was the prevailing genotype of DENV1 during the study period, with 86% of the sequences belonging to the Asia genotype. However, in 2014, 68% (65/96) of the DENV1 sequences belonged to the America/Africa genotype. Prior to 2014, only one and two sequences of the America/Africa genotype were detected in 2010 and 2013, respectively. Simultaneously, a major dengue fever epidemic occurred in 2014. Not all neutralizing antibodies stimulated by a certain genotype have binding and neutralizing activity against other genotypes^29,30^. This suggests that the neutralizing effect of antibodies to the Asia genotype may have a reduced inhibitory effect against the America/Africa genotype. Furthermore, although it is unclear whether some genotypes have a greater potential epidemiological impact than other genotypes, making them more likely to trigger outbreaks, there have been reports of outbreaks being accompanied by genotype shifts^31,32^. Therefore, the change in the dominant genotype from the Asia genotype to the America/Africa genotype might have been associated with the 2014 outbreak.

While the DENV1 Asia genotype remained the most common genotype, except in 2014, it could be further divided into sublineages I, II, and III. Most of the sequences were clustered in sublineage I in 2010, in sublineage III in 2011, and then returned to sublineage I from 2012 to 2014. Sequences from 2015 were distributed in all three sublineages. Most of the sequences from 2016 and 2018 belonged to sublineage I and shifted to sublineage II in 2019. However, these sublineage shifts were not associated with outbreaks.

Although DENV3 and DENV4 could not be detected every year throughout the study period, with far fewer sequences than DENV1 and DENV2, genotype shifts of DENV3 and DENV4 did occur. The DENV3 Thailand genotype was prevalent in 2012, but none of the sequences from other years belonged to this genotype. All DENV4 sequences in 2010 clustered in the Indonesia genotype, whereas all except one sequence in 2018 belonged to the Southeast Asia genotype. These genotype shifts were accompanied by changes in prevalence of the serotypes. DENV3 and DENV4 were the most prevalent serotypes in 2012 and 2010, respectively. This phenomenon, similar to DENV1, indicates that genotype shifts may be associated with changes in the incidence of dengue virus.

In contrast, the DENV2 Malaysia/India subcontinent genotype, which accounted for > 95% of the DENV2 sequences, and remained the predominant genotype throughout the study period, with no genotype shift, and was detected in almost every year. Even when the Malaysia/Indian subcontinent genotype was further divided into lineages 1 and 2, lineage 2 with 116 sequences remained the predominant lineage. Despite the persistent presence of DENV2 in Guangzhou, the lineages and genotypes did not shift during the study period, and DENV2 did not cause any obvious epidemic change. DENV2 reportedly transmits more readily and exhibits greater potential to cause severe dengue than the other three serotypes, especially if infection occurs following a heterotypic infection^33^. However, this ability varies among genotypes. Studies have revealed that the America genotype failed to cause severe dengue in Peru^34^, while the Southeast Asia genotype is more effective at causing infection and has greater potential to cause severe dengue^35,36^. Because the Southeast Asia genotype was rare in Guangzhou, the prevailing Malaysia/Indian subcontinent genotype appears to have limited capacity to cause severe dengue, which may explain the low incidence of severe dengue in China compared with other countries^26,27,37^. However, further studies are needed to determine whether the incidence of severe dengue is low in other areas dominated by the Malaysia/Indian subcontinent genotype and to determine the effects of different genotypes on the host immune system in order to improve the current understanding of the mechanisms of severe dengue.

Our study had some limitations. Not all dengue infections are reported, especially cases with mild symptoms that are difficult to diagnose. This may have led to the true dengue burden in Guangzhou being underestimated and to biased results. The analysis of the molecular characteristics of DENV was based on the sequences that we obtained. However, sequences were not obtained for all cases because serum samples could not be collected from all patients during the acute stage, and our technique was limited, especially before 2019; the analysis of the four serotypes is dependent on the data we were able to collect. We plan to continue to analyze as many sequences as possible in future years in order to improve our understanding of the epidemiology of dengue in Guangzhou. In this study, we attempted to identify connections between the genomic characteristics of DENV and the epidemiology of dengue cases in Guangzhou. Contrary to previous studies, there was no significant difference in the risk of infection between males and females, which may be attributable to the time-period of the study and the locally-acquired cases. The rising risk of infection with age revealed that elderly residents of Guangzhou were susceptible to DENV, and suggests that dengue had not been endemic in Guangzhou. Co-circulation of and shifts in the four serotypes occurred in Guangzhou during the study period, and genotype and sublineage shifts were also observed. Genotype shifts may have an epidemiological impact on dengue incidence; however, we did not find evidence of an association between shifts in serotypes or sublineages, and epidemics.

## Supplementary Information

Supplementary Information 1.

Supplementary Information 2.

## Data Availability

DENV sequences acquired in this study were deposited in the GenBank repository, https://www.ncbi.nlm.nih.gov/genbank/, with accession numbers shown in Table S3. The data that support the findings of this study are available from the corresponding author.
