# Supplementary material for: Epidemiological and genomic analysis of dengue cases in Guangzhou, China, from 2010 to 2019
Source: Sci Rep. 2023 Feb 7;13:2161. doi: 10.1038/s41598-023-28453-y (PMC9905598; doi:10.1038/s41598-023-28453-y)
Supplement: Supplementary file 2 — Supplementary Information 2. [file 41598_2023_28453_MOESM2_ESM.docx]

Epidemiological and genomic analysis of dengue cases in Guangzhou, China, from 2010 to 2019

Jiang et al.

**Table S1. Primers used to amplify and sequence E gene of dengue virus.**

| **Virus** | **Primer** | **Sequence** |
| --- | --- | --- |
| DENV1 | DEN1 F | 5'-CTCTGAGACACCCAGGATTCAC-3' |
|  | DEN1 R | 5'-GCTGATCGAATTCCACACAC-3' |
| DENV2 | DEN2 F | 5'-CCAGGCTTTACCATAATGGC-3' |
|  | DEN2 R | 5'-CCAGCTGCACAACGCAACCAC-3' |
| DENV3 | DEN3 F | 5'-AGTCGAGAAGTAGAGACATGG-3' |
|  | DEN3 R | 5'-CTCTGTCCAGGTGTGGACCT-3' |
| DENV4 | DEN4 F | 5'-CTGTTTTCTTTGTCCTAATGATGCT-3' |
|  | DEN4 R | 5'-AACCCATGTCTGCTTGAACTGTGA-3' |

* DENV, dengue virus

**Table S2. Age-sex standard incidence rates**

| **Sex** | **2010** | **2011** | **2012** | **2013** | **2014** | **2015** | **2016** | **2017** | **2018** | **2019** |
| --- | --- | --- | --- | --- | --- | --- | --- | --- | --- | --- |
| **Male** | 0.786 | 0.387 | 1.098 | 9.027 | 282.192 | 1.108 | 2.072 | 7.278 | 10.601 | 13.394 |
| **Female** | 0.682 | 0.533 | 1.359 | 10.880 | 310.238 | 1.008 | 2.138 | 7.237 | 8.901 | 12.397 |

* Per 100,000 population

**Table S3.** **The accession numbers of DENV sequences acquired in Guangzhou between 2010 and 2019**

| serotype | accession number of each year | | | | | | | | | |
| --- | --- | --- | --- | --- | --- | --- | --- | --- | --- | --- |
|  | 2010 | 2011 | 2012 | 2013 | 2014 | 2015 | 2016 | 2017 | 2018 | 2019 |
| DENV1 | MT804414,MT804415,MT804421,JN009086, JN009087, JN009089 | JX088739, JX088740, JX088742, JX088743,MT804427 | KF060913-KF060918 | KR870408-KR870422,MT804428-MT804458 | KR006700-KR006728,MT804459-MT804525 | MF381070-MF381079 | MN933660-MN933663,MN933691-MN933715 | MN933664,MN933672-MN933690,MT804526-MT804546 | MN933716-MN933766 | MN921254-MN921303, MN921305-MN921313, MN921315, MN921317-MN921319, MN921321,MN921322, MN921324-MN921333, MN921335-MN921403, MN921405-MN921417, MN921419-MN921475, MN921478-MN921586, MN921588-MN921601, MN921603-MN921631, MN921633-MN921642, MN921644,MN923077, MN923080, MN923082-MN923086, MN923088-MN923106 |
| DENV2 | JN009091, JN009092 | None | KF060919,KF060920 | KJ807797,MT845880 | KR029565-KR029570,KR071787,MG593022-MG593024 | KU051419,KX027278,KX027279,MG593025-MG593030 | MG596004-MG596027 | MG677986-MG678001 | MN923132-MN923170 | MN915156,MN915157, MN915159-MN915163, MN915165-MN915169, MN915171-MN915189, MN915191-MN915197 |
| DENV3 | JN009093-JN009098 | None | KY673718-KY673726,MT860495-MT860500 | None | None | KY673727 | KY673728 | None | MK894338-MK894341 | MN915198-MN915219 |
| DENV4 | MT835124-MT835137 | None | KC333651 | None | None | None | MT835138 | MW295825 | MK614090-MK614093,MK640208 | MK614088 |
