# Supplementary material for: Epidemiological and genomic analysis of dengue cases in Guangzhou, China, from 2010 to 2019
Source: Sci Rep. 2023 Feb 7;13:2161. doi: 10.1038/s41598-023-28453-y (PMC9905598; doi:10.1038/s41598-023-28453-y)
Supplement: Supplementary file 1 — Supplementary Information 1. [file 41598_2023_28453_MOESM1_ESM.pdf]

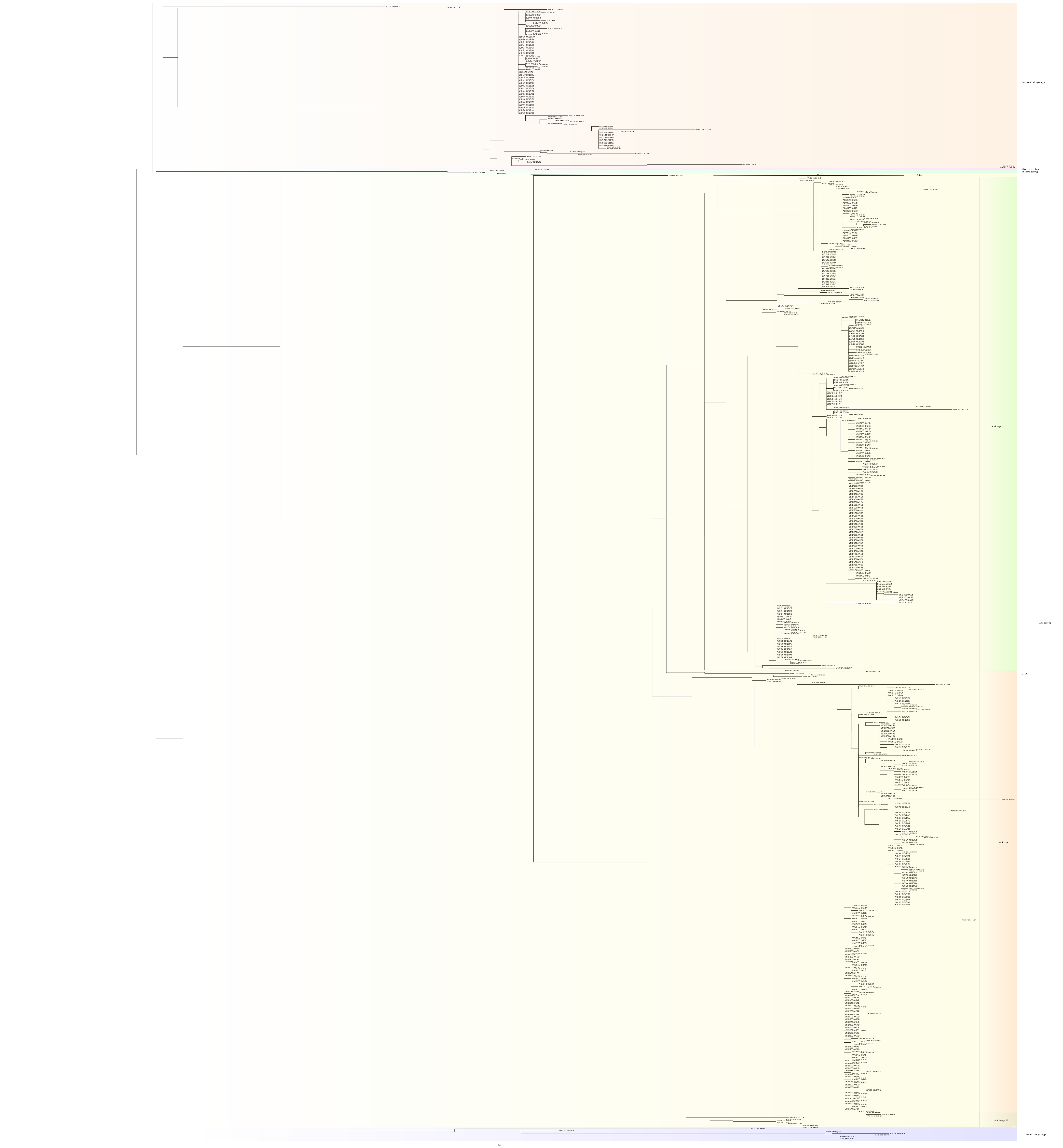

Supplementary figure 1. Detailed phylogenetic trees of DENV1 E gene sequences.
